# From Preferred to Actual Mate Characteristics: The Case of Human Body Shape

**DOI:** 10.1371/journal.pone.0013010

**Published:** 2010-09-27

**Authors:** Alexandre Courtiol, Sandrine Picq, Bernard Godelle, Michel Raymond, Jean-Baptiste Ferdy

**Affiliations:** 1 CNRS – Institut des Sciences de l'Evolution, Université Montpellier 2, Montpellier, France; 2 CNRS – Evolution et Diversité Biologique, Université Paul Sabatier – Toulouse III, Toulouse, France; Smithsonian Institution National Zoological Park, United States of America

## Abstract

The way individuals pair to produce reproductive units is a major factor determining evolution. This process is complex because it is determined not only by individual mating preferences, but also by numerous other factors such as competition between mates. Consequently, preferred and actual characteristics of mates obtained should differ, but this has rarely been addressed. We simultaneously measured mating preferences for stature, body mass, and body mass index, and recorded corresponding actual partner's characteristics for 116 human couples from France. Results show that preferred and actual partner's characteristics differ for male judges, but not for females. In addition, while the correlation between all preferred and actual partner's characteristics appeared to be weak for female judges, it was strong for males: while men prefer women slimmer than their actual partner, those who prefer the slimmest women also have partners who are slimmer than average. This study therefore suggests that the influences of preferences on pair formation can be sex-specific. It also illustrates that this process can lead to unexpected results on the real influences of mating preferences: traits considered as highly influencing attractiveness do not necessarily have a strong influence on the actual pairing, the reverse being also possible.

## Introduction

The way individuals pair to produce reproductive units is a major factor determining evolution. First, this pairing process can modify allelic frequencies through sexual selection: alleles that increase the probability that the carrier is chosen as a mate are, *ceteris paribus*, positively selected [e.g. 1]. Second, the pairing process can influence several important genetic parameters [2]. For instance, assortative mating can increase additive genetic variance, which in turn increases response to selection [e.g. 3]. Finally, the pairing process has been shown to affect various demographic aspects such as survival rates, population size, or sex-ratios [4], which are also key components in the evolution of populations. At a larger scale, the pairing process can therefore, directly or indirectly, lead to phenotypic modification of organisms [5]; it can also affect extinction rates of populations [6] and it could play an important role in speciation [e.g. 7].

Mating preferences are an important aspect of the pairing process. That is why researchers often deduce mating preferences from outcomes of traditional pairwise mate choice experiments [8], [9]. Nevertheless, in more natural contexts, many other factors besides mating preferences are also involved in the process of pair formation (reviewed in [10]–[13]). Partners' availability, sampling strategies, competition within one sex, coercion, environmental influence on mate assessment, or preferences exerted by the other sex (when mate choice is mutual), are examples of other factors apart from preferences that can have a major influence on the outcome of pair formation. Consequently, even under very simple rules determining the pairing process, the link between preference and pairing outcome is generally not straightforward [14], [15]. For instance, in simulations where only the way in which sexes encountered each other is varied, the same preference rule yields either homogamous mating patterns (when all pairs are formed simultaneously) or heterogamous mating patterns (when pairs are formed sequentially [14]). Overall, despite the large body of research on mate choice, understanding how preferences translate into real pair formation appears to be a challenging, and surprisingly neglected, problem.

To understand how mating preferences translate into observed mating patterns, an important step would be to measure mating preferences, and to compare them with actual pairing involving the same individuals *in natura*. This could be empirically challenging for many animal species, but it remains feasible in humans. In addition, both mating preferences and mating patterns have been extensively studied in this species. In particular, traits related to body shape have interested a large panel of scientists working in different fields including anthropology, evolutionary biology, demography, economy, medical sciences, psychology and sociology [e.g. 9], [16]–[29]. In this literature, almost all studies concern either mating preference or mating patterns, independently (but see [30], [31]). Yet, as in numerous studies focussing on mate choice in other animals, authors often implicitly assume a direct causal relationship between mating preferences and mating patterns.

The present study is the first attempt to compare preferences and actual mate characteristics concerning body shape in both men and women. We specifically address this question for Stature (S), Body Mass (BM), and their combination BM/S^2^ which defines the Body Mass Index (BMI). Indeed, these variables have been shown to explain most of inter-individual variation in body shape [32] and they are known to be important traits regarding mating preferences in both sexes [e.g. 9], [20], [21], [28], [29], [33]. To do this, we measured mating preferences and recorded mate characteristics for both partners in 116 couples sampled in Montpellier (France). We firstly estimated preferences of each individual by using a software in which one can directly manipulate the body shape (stature and BMI) of a virtual silhouette to represent his/her preferences. Then, we compared body shape characteristics obtained for these preferred silhouettes with the corresponding characteristics of actual partners. We also used the same software to examine self-representation of body shape to ensure that our method to estimate preferences had not been flawed by potential cognitive biases.

## Materials and Methods

### Ethics Statement

Informed oral consent has been obtained from each participant. All participants received a written notice explaining that their participation was optional and that they could refuse to answer any question during the experiment. Anonymity and confidentiality of subjects were guaranteed at all stage of the study. Each participant was also given an anonymous number so that we could access, modify or suppress any information present in the database by simple request. All steps of the present study were approved by the ‘Commission Nationale de l'Informatique et des Libertés’ (registration # 1261003). This national commission has the general mission of ensuring that the development of information technology remains at the service of citizens and does not breach human identity, human rights, privacy or personal or public liberties (for more information, see www.cnil.fr).

### Methods

We conducted a study to compare the preferred stature, body mass and BMI of each individual with that of his/her actual partner. To do so, we sampled 116 couples in public areas of Montpellier (France) during spring 2008. To reduce culturally based variation in preferences, we did not consider people with any non-European grandparent, leading to a final dataset of 96 couples. In this dataset, median relationship duration was 36 months (mean ± SD: 59±63.1, range: 1–360). For females, median age was 25.8 years (mean ± SD: 27.3±7.4, range: 18.3–53.1), median stature was 165.0 cm (mean ± SD: 165.1±6.3, range: 151.0–178.0), median body mass was 57.0 kg (mean ± SD: 59.2±9.5, range: 45–90) and median BMI was 21.0 kg.m^−2^ (mean ± SD: 21.7±3.0, range: 14.9–30.4). In males, median age was 27.6 years (mean ± SD: 28.6±7.2, range: 18.6–50.6), median stature was 177.2 cm (mean ± SD: 177.5±6.3, range: 164–195), median body mass was 73.0 kg (mean ± SD: 74.6±9.8, range: 55–100) and median BMI was 23.2 kg.m^−2^ (mean ± SD: 23.7±3.1, range: 18.3–32.6). All these measures were obtained during an enquiry occurring just before the experiment, and therefore correspond to self-reported data.

**Figure 1:**
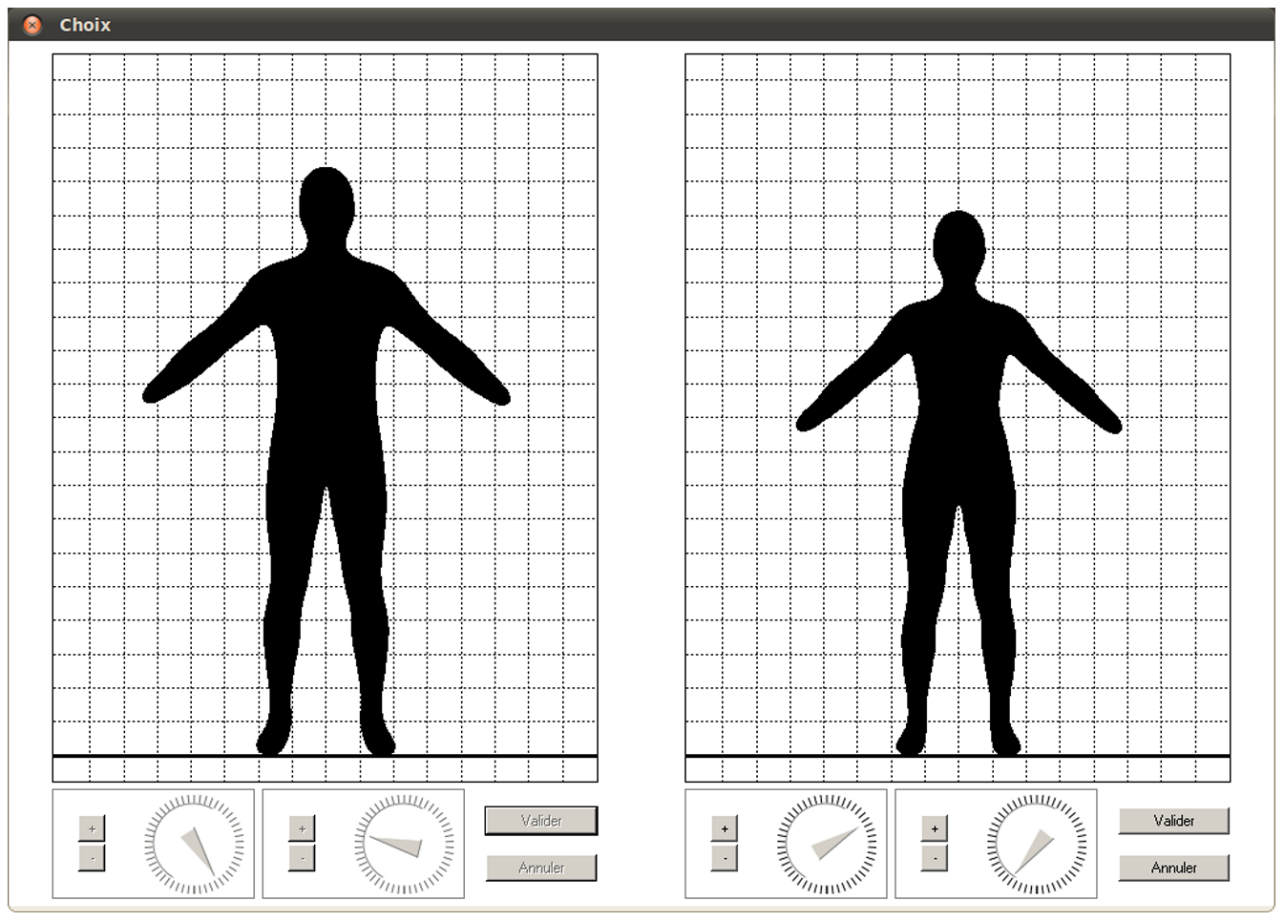
Snapshot of the software used to measure male preferences for body shape. The left picture represents a silhouette corresponding to the male (here: stature  = 183.0 cm, BMI  = 23.6 kg/m^2^), and the right picture represents the silhouette of his ideal female partner (stature  = 169.3 cm, BMI  = 18.3 kg/m^2^). Dials under the pictures enable the user to modify the BMI according to his self perception, and it enables him to adjust the stature and body mass of his preferred female as well. Whether the silhouette of the focal individual appears on left or right panel is randomly decided for each individual. The dial controlling the stature of the focal individual is inactivated, but is displayed for symmetry.

To estimate preferences we designed software that presents silhouettes for which stature and BMI can be manipulated independently (figure 1). The silhouettes have been derived from real pictures using a modelling method that allowed us to alter both stature and BMI while keeping the overall proportions of the silhouette realistic (see [34], for details). For each couple, the male was firstly presented a silhouette of his gender and stature. He was then asked to adjust the silhouette to resemble his body type. To do this, he had to modify the BMI using a virtual dial. This step enabled us to estimate the subject's self-representation of BMI, which can be biased by self-perception disorder [35] or by difficulties in identifying oneself with a virtual representation. After the focal male individual had manipulated the reference silhouette, a female silhouette was displayed next to it. This latter silhouette had a randomly chosen stature and BMI (range for stature: 156–176 cm, range for BMI: 16–38 kg.m^−2^). The focal individual was asked to change the morphological aspects of the female silhouette until it represented his ideal partner by turning dials controlling stature and BMI. A similar procedure was performed for the female of the couple: she first had to manipulate a female silhouette to measure her self-perception bias for BMI, and then she had to manipulate a male silhouette to represent her preferences (range for stature: 159–190 cm, range for BMI: 19–35 kg.m^−2^). The software for shape modification contains 399 999 different silhouettes for each sex, so that shape manipulation should have been perceived as continuous by the user. In addition, individuals were never informed about the role of the dials they had to manipulate and were, during all sessions, separated from their partner to prevent any influence on their preference estimation.

To compare preferences and actual characteristics of mates for a given sex, we firstly studied the distributions of differences between preferred and partner's values for each trait studied: stature, body mass and BMI. As the extent of variation in morphological traits can be influenced by their absolute values, to compare scales of these distributions we also performed these comparisons after having standardized differences between preferred and partner's values by dividing them by the standard deviation of partner's values in each sex. Finally, to study association between preferences and actual partner's characteristics, we performed a linear regression model for each trait. For stature, the response variable considered in the models was the partner's stature and we included the preferred body mass and the preferred stature as two covariates. Taking into account the preferred body mass as a covariate enables us to study the direct relationship between preferred and partner's stature controlling for indirect effect due to the anatomical relationship between stature and body mass. Similarly, for body mass, the response variable considered was the partner's mass and the two covariates included were the preferred stature and the preferred body mass.

For BMI, the partner's BMI could be influenced by direct preferences for BMI i.e. preferences for a certain body shape independently of preferences for stature and body mass, or by preferences for stature and body mass which generate indirect preferences for BMI. We built a first model including partner's BMI as a response variable and with a unique covariate corresponding to direct preferences for BMI. The direct preferences for BMI correspond to the BMI of the silhouette built by the subject in accordance with his/her preferences. A second model for BMI, based on the previous one, includes the indirect preferences for BMI as an additional covariate. The indirect preferences for BMI were built using predicted partner's stature and body mass obtained through corresponding models previously described, that were combined following the BMI formula. This second model allows the examination of the relationship between direct preferences for BMI and partner's BMI independently of BMI preferences caused by preferences for stature and body mass.

For all regression models, normality of residuals was tested by the Shapiro-Wilk test, while homoscedasticity and independence of residuals were tested using the Breusch-Pagan test and the Durbin-Watson test, respectively. In cases these assumptions are violated even when potential outliers are removed (outliers being detected using the Bonferroni outlier test), Box-Cox transformations were performed, after which assumptions were met in all instances. All data analyses were performed under R 2.9.2 (http://www.R-project.org) using the car [36], lmtest [37] and MASS packages [38].

## Results

Our study consisted of asking individuals to manipulate the body shape of virtual stimuli to represent their mating preferences. Women preferred an ideal partner with a median stature of 178.4 cm (mean ± SD: 178.9±6.3, range: 161.3–189.7), a median body mass of 74.5 kg (mean ± SD: 75.7±11.4, range: 51.8–109.0) and a median BMI of 23.5 kg.m^−2^ (mean ± SD: 23.6±3.1, range: 19.0–33.7). Men preferred an ideal partner with a median stature of 166.8 cm (mean ± SD: 166.5±5.1, range: 156.0–175.8), a median body mass of 52.0 kg (mean ± SD: 53.0±6.9, range: 39.7–77.2), and a median BMI of 18.4 kg.m^−2^ (mean ± SD: 19.1±2.4, range: 16.0–27.4). The degree of variation in preferences did not differ between sexes (two-sample Ansari-Bradley tests, for stature: *AB*  = 4806, *p* = 0.44; for body mass: *AB*  = 4663, *p* = 0.97; for BMI: AB  = 4435, *p* = 0.25) and this also remains true if preferences are standardized by standard deviations of actual partners' values for each trait within sexes (for all tests *p*>0.2).

**Figure 2:**
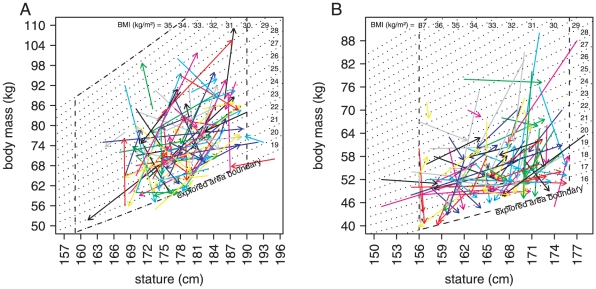
Preferences and partners' body shape characteristics for females (A) and males (B). Each arrow represents information for one individual. The start of an arrow represents the partner's characteristic and the end represents preferences measured for the same individual. Dotted lines represent combinations of stature and body mass which correspond to equal BMI. The areas delimited by dashed lines (labelled explored area boundary) represent body shape characteristics available for building preferred silhouettes. Colours are displayed for a graphical purpose only.

Individual preferences can be compared to actual partner's characteristics of these individuals in figure 2. For women, medians of differences between preferences and actual partner's characteristics of these women were +0.99 cm (mean ± SD: +1.35±8.01, range: −17.65 − +25.22) for stature, +1.41 kg (mean ± SD: +1.03±13.24, range: −34.86 − +36.70) for body mass, and +0.06 kg.m^−2^ (mean ± SD: −0.09±3.97, range: −13.60 − +9.39) for BMI. On average, none of these differences statistically differ from zero (table 1). For men, medians of differences between preferences and actual partner's characteristics of these men were +0.45 cm (mean ± SD: +1.37±5.94, range: −11.00 − +20.00) for stature, −5.12 kg (mean ± SD: −6.24±9.52, range: −37.94 − +13.03) for body mass, and −2.44 kg.m^−2^ (mean ± SD: −2.58±2.79, range: −12.09 − +3.91) for BMI. On average, males preferred females marginally taller, significantly lighter and with lower BMI values than their partners (table 1). To compare the scale of differences between preferences and actual partners' characteristics between sexes, we standardized these measures by dividing them by standard deviations of partners' values for each trait within sexes (see methods). For all traits, the degree of variation in these standardized differences are much higher in females than in males (two-sample Ansari-Bradley tests, for stature: *AB*  = 5215, *p<*0.004; for body mass: *AB*  = 5233, *p<*0.003; for BMI: *AB*  = 5167, *p*<0.008). Again, similar conclusions are obtained if the standardization is not performed (for all test *p*<0.006). Importantly, the duration of the relationship does not bias these conclusions since the scale of differences between preferences and actual partners' characteristics do not differ between a sub-sample including only couples with a relationship duration less than the median value and a sub-sample including only couples with a relationship duration equal or superior to 3 years (all *p*>0.19).

**Table 1:** Comparison between preferences and actual partner's characteristics.

Trait	Gender of the chooser	Median		Median		Difference			Association			
		Preferred value		Partner's value		*W_+_* a		*p* b	*F* c		*p* e	Model *r* ^2^ f
Stature	Female	178.4	cm	177.2	cm	2760		0.12	3.9		0.051	0.04
–	Male	166.8	–	165.0	–	2810		0.079	28	<	0.001	0.24
Mass	Female	74.5	kg	73.0	kg	2397		0.80	3.1		0.083	0.05
–	Male	52.0	–	57.0	–	759	<	0.001	13	<	0.001	0.13
BMI	Female	23.5	kg/m^2^	23.2	kg/m^2^	2292		0.90	4.0		0.047	0.04
–	Male	18.4	–	21.0	–	334	<	0.001	29^d^	<	0.001	0.24

(*a*) Statistics of the Wilcoxon's signed rank test used to compare location of preferred and partner's trait values.

(*b*) P-values of the Wilcoxon tests.

(*c*) Fisher's statistics which indicate the strength of the associations between preferred and partner's trait value (Nb: all *F* have the same numerator and denominator degrees of freedom: 1 and 93, respectively; except for the model for female preferences for BMI for which the denominator degrees of freedom is 94).

(*d*) One outlier has been removed from the dataset for this model (see figure 3).

(*e*) P-values of the associations.

(*f*) Proportion of variance explained by the regression models.

Another way of looking at the relationship between mating preferences and actual partner's characteristic is to study the associations between these variables. In order to study the associations between mating preferences and partner's characteristics, we used linear models (see methods). Model summary statistics are given in the last three columns of table 1. Results show that there is weak correlation between the body shape characteristics preferred by females and the actual characteristics of the partners of these females (figure 3a,c,e). In contrast, male preferences for stature, body mass and BMI correlate well with their partners' characteristics (figure 3b,d,f).

**Figure 3:**
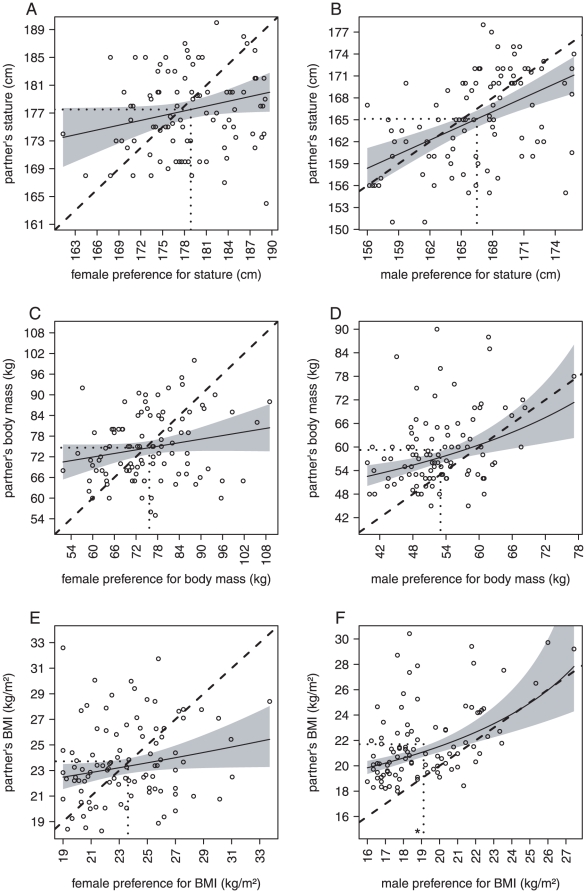
Plots of regression models between preferred and actual partner's characteristics. Plots of models concerning females and males subjects are presented on the left and on the right, respectively. The first row represents plots of models for stature, the second row for body mass, and the last for BMI (considering only indirect preferences, see text for details). Grey shaded area represents 95% confidence interval of the regression lines. Dashed straight lines represent perpendicular bisectors (*y = x*). Dotted lines represent mean values. Data of the models represented in plots D, E and F have been transformed using Box-Cox transformations to reach linear model assumptions. The star label in the plot F represents data of an outlier not considered in the statistical analysis.

The actual partners' BMI can be predicted either from the preferred BMI (direct preference), or by using the preferred stature and body mass to compute a preferred BMI (indirect preference, see methods). Table 1 indicates only the direct preference model. Nonetheless, direct and indirect preferences for BMI are highly correlated (*ρ* = 0.99), and using indirect preferences alone to predict the actual partners' BMI could be sufficient. In fact, it appears that considering direct preferences for BMI in a model that already includes indirect preferences does not improve the quality of the prediction for either sex (for females: *F*_1,93_ = 0.01, *p* = 0.91; for males: *F*_1,92_ = 0.09, *p* = 0.76).

Importantly, our methodology to assess preferences assumes that individuals have correctly perceived the body shape of the displayed silhouettes. In order to check this assumption we compared the self-perceived BMI, obtained through the manipulation of the silhouette representing individuals whose preferences are examined, to the actual BMI, reported by individuals. For both sexes, perception biases in BMI represent less than 25% of the actual BMI for 94% of individuals sampled, and perceived and actual BMI are highly correlated (*ρ*>0.7). Moreover, the best adjusted regression line between perceived and actual BMI does not statistically differ from a line of origin zero and of slope one (model comparison: *F*_2,190_ = 26, p = 0.26), meaning that there is no directional self-perception bias in our population. Correcting preferences obtained for body mass or BMI by taking into account individual self-perception biases (either additively or multiplicatively) led to very similar results. In addition, for couples that were formed several years before this study, people may have gained body mass since the time they paired with their partner. Indeed, BMI is known to increase within the age range we considered [39]. Unfortunately, we do not know how much weight each individual put on since the beginning of the relationship, but in order to examine roughly this possibility we also performed our analyses correcting for the average change in the partner's BMI and body mass during the relationship (based on predictions obtained from regressions of BMI against age in our cross-sectional dataset). Again, results obtained with this correction led to qualitatively similar conclusions.

## Discussion

The literature focusing on mating preferences or mating patterns is particularly vast with regards to body shape in humans. There is indeed a long lasting tradition of studying the influence of human stature in mating pattern, starting from the end of the 19^th^ century [e.g. 18], [40]–[42]. Surprisingly, there are only few studies measuring preferences for stature [43], but recent publications demonstrate a renewed interest in this matter [9], [33], [44]. In addition, the recent worldwide increase of obesity has encouraged research focussing on mating preferences for BMI, and there is now a large body of evidence demonstrating that BMI is an important determinant of attractiveness in both men and women [20], [21], [28], [29]. Several studies have also demonstrated that paring is non-random with respect to BMI [e.g. 18], [45], even though departure from random mating seems lower than the one observed for stature [27]. Overall, in these studies, the two ultimate key questions researchers attempt to answer are: (i) how do mating preferences influence the evolution of morphology, and (ii) how do selective pressures shape mating preferences? Nevertheless, we strongly believe that to answer these questions, it is indispensable to also address a third and completely overlooked question, namely: how do the mating preferences influence the actual outcome of the pairing process *in natura*?

Thus, we measured mating preferences for stature, body mass and BMI, in 96 males and 96 females involved in a relationship, and we compared these preferences with the actual body shape characteristics of their partners. Our results show that on average female preferences did not differ from body shape characteristics of their male partners. However, the average body shape preferred by males differed from the actual morphology of their female partners for the three characteristics studied: men preferred women marginally taller, lighter and thinner than their female partners. In particular, men preferred BMI values close to the cut-off value of 18.5 kg.m^−2^ used by the World Health Organisation to delimit normal and underweight BMI (http://apps.who.int/bmi/index.jsp?introPage=intro_3.html), while average BMI of women in our sample is around 22 kg.m^−2^. This bias in male preferences is therefore high, but as obese individuals (BMI >30 kg.m^−2^) make up just 2% of the present sample, we would expect this difference between male preferences and actual female characteristics to become even more important in the context of the current rise in obesity (if BMI values preferred by males remain the same). Our methodology for estimating preferences can also be used to study preferences for stature and BMI independently. Using this property, we showed that the association between mating preferences for BMI and actual partner's BMI can be very well approximated by using mating preferences for stature and body mass, meaning that individuals that have the same BMI but different statures and body masses are not equally preferred. Therefore, the male preferences for low BMI correspond to preferences for females taller and lighter than their actual partners (rather than reflecting a direct preference for BMI). Importantly, our methodology does not seem to bias the estimation of mating preferences since estimated preferences for BMI are qualitatively similar to preferences reported in other studies that rely on other protocols [e.g. 21], [46]. Concerning stature, although not significant, our results show similar tendencies to the ones reported in previous studies ([9] and references therein).

In addition, we observed an important difference between what individuals prefer and what they actually get in both sexes. Indeed, even within females, for which differences between ideal and actual partner's body shape are null on average, the large variance characterizing the distributions of these differences illustrates a high mismatch between preferred and actual partner's characteristics for a given individual. In fact, the correlation between preferences and actual partner's characteristics is much higher in males than in females for the three body shape characteristics (as represented by figure 3). For instance, only 4% of the total variance in partners' BMI is explained by female preferences for this trait, while for males whose preferences for stature and body mass together explained 26% of the variability in their partners BMI. Note that the high correlation between male preferences for BMI and their partners' BMI is not incompatible with male biased preferences for low BMI values. Indeed, our results suggest that men prefer women slimmer than their actual partner; but still, men who prefer the slimmest women also have partners who are slimmer than average.

The strongest associations between mating preferences and actual partner's characteristics observed for males could be due to several distinct phenomena. First, it has been argued that females place less emphasis on physical attractiveness than males [47]. Hence, if females base their choice on traits that are not perfectly correlated to the physical traits we measured (e.g. socioeconomic status), we expect the correlation between their preference and their mates' physical characteristics to be lower than the same relationship measured in males. Second, males might have a much stronger influence on the outcome of mate choice. In that case, we would expect to see a stronger relationship between preferences and partner's characteristics for males than for females if preferences on both sides lead to a disagreement about the mate choice. Such disagreement has already been demonstrated for stature [9], but it is likely to be a frequent feature of mutual mate choice situations. Replicating the present study but focusing on other traits that females might choose could allow us to distinguish between these two hypotheses. Nonetheless, if the male influence on mate choice is only slightly stronger than the female, both hypotheses could apply. Different patterns of preference versus reality can indeed be predicted depending on the control each sex has on how mates are chosen.

In addition, certain processes of pair formation can give an important advantage to one sex despite the mutuality of the choice. For instance, in the Gale and Shapley [48] algorithm of stable pair formation (which is widely used in economic sciences), a well known property is that individuals of the sex that courts (here, it would be the males) better translate their preferences than the sex that exerts the final choice between potential mates [49]. Identifying actual pairing processes operating in human populations seems particularly difficult, but replicating this study in human populations for which mate choice is not mutual should bring decisive elements to better understand the observed sex discrepancy.

Another potential explanation of the weak correlation between preference and actual partner's characteristics in females could be that females almost always prefer the same type of partners. However, we found no difference between variability in male preferences and variability in female preferences, meaning that this hypothesis is not likely. Indeed, if this hypothesis was correct, the variability in preferences observed in females would then correspond to measurement errors rather than to true differences in preference. In that situation, the differences between measured preferences would not be expected to translate into a difference between chosen partners. Still, as these measurement errors also exist in males and as we found evidence that not all males have the same preferences, we would expect a higher variability in preferences in males than in females.

Finally, we are also aware that the association between preferences and actual partner's characteristics does not necessarily demonstrate that preferences influence partner choice. The alternative is to consider that people adjust their preference to reflect the characteristics of their partner. As correlations do not allow us to infer causation, we cannot evaluate this possibility. Still, the correlation between actual and preferred partner's characteristics is not influenced by the duration of the relationship (data not shown) as would be expected if males were adjusting their preferences. In addition, the adjustment of preferences would not explain why male preferences are more influenced by their partner's characteristics than females, especially given that average preferences differed from average actual trait values for males and not for females.

Although this study concerns a limited number of traits and a single species, it illustrates the complexity of the relationship between preferences and actual pairs. Indeed, our results suggest that some traits that are considered as highly influencing attractiveness do not necessarily have a strong influence on the outcome of the pairing process. For instance, we showed that female preferences for stature and BMI appear to be poor predictors of their partners' body shapes, although these traits are considered as relatively good predictors of attractiveness [e.g. 29], [46], [50]. Conversely, female's stature is not considered as an important attractiveness component in the literature but we demonstrate here that it strongly correlates with male preference, which therefore suggest that this trait could play an important role in pair formation.

To conclude, these findings illustrate the fact that little can be predicted about mating patterns from the simple observation of mating preferences, and reciprocally, little can be predicted about mating preferences from the simple observation of mating patterns. This comes from the fact that the way couples are formed depends on several processes that have never, or only rarely, been studied. In particular, we demonstrate that in a species where mutual mate choice occurs, the pairing process can lead to large asymmetries in the expression of mating preferences between sexes. For instance, we observed that variation in differences between preferred and partner's characteristics differ between females and males. Such asymmetries could potentially lead to complex evolutionary consequences through the development of different selective pressures for each sex acting on preferences and ornaments. This result is of particular interest given that there has been a recent accumulation of evidence suggesting that even in systems traditionally described as one side mate choice, members of the other sex are often not completely indiscriminate [51]–[53]. A lot of work remains to be done to unravel issues raised by pair formation processes. This includes theoretical studies that are necessary to better understand how preferences translate into choice in a context of competition and mutual choice. This also includes detailed studies of the pair formation mechanisms in various natural systems, including humans. We hope that this paper will stimulate work in all these directions.
